# Combination therapy with low molecular weight heparin and Adriamycin results in decreased breast cancer cell metastasis in C_3_H mice

**DOI:** 10.3892/etm.2014.1911

**Published:** 2014-08-18

**Authors:** WEIWEI YIN, JING ZHANG, YUMIN JIANG, SHEN JUAN

**Affiliations:** Department of Nursing, Bengbu Medical College, Bengbu, Anhui 233030, P.R. China

**Keywords:** low molecular weight heparin, C3H mice, breast cancer

## Abstract

The aim of this study was to investigate the effect of low molecular weight heparin (LMWH) against breast cancer cell invasion and metastasis in C_3_H mice and the underlying mechanism. The C_3_H mouse breast cancer model was established, and the mice were then randomly divided into four groups: normal saline group, LMWH group, Adriamycin positive control group and the combination group (LMWH combined with Adriamycin). Twelve days after inoculation, drug treatment was initiated. During the one-month period of drug administration, tumor growth curves were recorded. At the end of the treatment period, the mice were sacrificed and the solid tumor tissue and lung were removed. Hematoxylin and eosin (H&E) staining was used to observe the overall changes in tumor cell morphology and lung metastasis following the treatment. A terminal-deoxynucleotidyl transferase-mediated nick end labeling (TUNEL) assay was used for detection of apoptosis in tumor cells, and immunohistochemical (IHC) analysis was used to determine the expression of vascular endothelial growth factor (VEGF). The tumor growth curves demonstrated that the overall growth of the combination group was less compared with that of the other three groups, indicating that LMWH inhibited the growth of the tumors. H&E staining showed that the area of tumor cell necrosis in the combination group was significantly greater compared with that in the other groups, and less metastasis was observed in the lung. The results from the TUNEL staining demonstrated that there was an increase in the number of blue-black apoptotic cells, and the expression of VEGF was significantly reduced in the combination group compared with the other three groups. Therefore, this indicates that LMWH, combined with Adriamycin significantly reduced the growth of breast cancer cells in C_3_H mice. The results suggest that the mechanism may be associated with breast cancer cell apoptosis and inhibition of VEGF expression.

## Introduction

Breast cancer is the most common type of malignant tumor and a leading cause of cancer mortality. At present it has the highest incidence of malignant tumors in females (1). Approximately 200,000 females per year are diagnosed with breast cancer and breast cancer results in 40,610 mortalities in the USA (2). The current treatment may involve lumpectomy (surgical removal of the tumor with clear margins) or mastectomy (surgical removal of the breast), as well as chemotherapy, radiotherapy and other therapeutic treatments. Among these, chemotherapy has the most important role. Adriamycin is commonly used since it has a broad-spectrum antitumor effect. However, it also has a strong cytotoxic effect, including bone marrow suppression.

Heparin has long been known to possess biological effects that are not associated with its anticoagulant activity, and it is the first choice for the prevention and treatment of venous thromboembolism for patients with cancer. In particular, heparin and novel agents based upon the heparin template have been investigated as potential antitumor agents. Previous studies have suggested that heparin, as well as having direct effects on blood coagulation, also has a role in the treatment of cancer by inhibiting tumor cell proliferation, angiogenesis, invasion and migration, and enhancing the sensitivity of tumor cells to chemotherapeutic drugs (3–5). The possible specific mechanisms thought to be involved in the antitumor effect of heparin include inhibition of heparanase activity and inhibition of the tissue factor pathway (6).

It was hypothesized in the present study that combination treatment with small doses of Adriamycin and heparin would lead to reduced tumor angiogenesis, and in turn metastasis, without major adverse effects. In this study, a breast cancer model in mice was established and the mice were randomly divided into four groups, treated with Adriamycin or heparin alone, a combination of Adriamycin and heparin or saline. All mice were analyzed for tumor weight, metastasis, vascular endothelial growth factor (VEGF), microvascular density (MVD) and overall quality of life. The tumor metastasis rate and occurrence of adverse effects in the various groups were compared, and the mechanism of action was investigated. Analysis of the results was conducted to evaluate whether heparin and Adriamycin combination treatment may be a novel strategy for the treatment of breast cancer.

## Materials and methods

### Cell line and culture

C_3_H mouse autologous breast cancer cells were obtained from Bengbu Medical College (Bengbu, China). Cells were grown in Dulbecco’s modified Eagle’s medium (DMEM), supplemented with 10% fetal calf serum, penicillin (10 U/ml), streptomycin (100 U/ml) and HEPES (25 mM), and maintained at 37°C in a humidified atmosphere of 5% CO_2_.

### Experimental animals and grouping

A total of 40 healthy, female C_3_H mice, weighing 17–22 g, were provided by Silaike Experimental Animals Co., Ltd. (Shanghai, China). The mice were randomly divided into four groups (n=10/group): normal saline group, low molecular weight heparin (LMWH) group, Adriamycin positive control group and a combination group (LMWH combined with Adriamycin). All animals were examined prior to the start of the study and any animal that did not meet the health and weight criteria was excluded from the study. This study was performed in strict accordance with the recommendations in the Guide for the Care and Use of Laboratory Animals (8th edition, 2011) of the National Institutes of Health (Bethesda, MD, USA). The study was approved by the Ethics Committee of Bengbu Medical College.

### Establishment of a breast cancer model in C_3_H mice

Exponentially growing tumor cells (0.2 ml; 1×10^7^/ml) were subcutaneously injected into the right axillary region of C_3_H mice. Tumor lines were achieved by serial subcutaneous passages of tumor fragments (~3×3×3 mm) from growing tumors into C_3_H mice, as previously described (7).

When the tumor volume was the size of a millet seed, this indicated that a breast cancer model was successfully established in the C_3_H mice. The normal saline group was administered saline intraperitoneally, once daily (1 ml/20 g). The Adriamycin group was administered Adriamycin (Xing Jia Biological Medicine Co., Ltd., Wuhan, China) intraperitoneally, once weekly (4 mg/kg/dose). The LMWH group was administered LMWH (Wang Bang Biosciences Co., Ltd., Xuzhou, China) subcutaneously, once daily (1,500 U/kg/day). The combination group was treated with Adriamycin and LMWH, at the same doses as for the Adriamycin and LMWH groups. Treatment was administered for one month. Tumor growth was followed by biweekly measurements of tumor diameters using a Vernier caliper 12 days following inoculation. Tumor volume (TV) was calculated according to the following formula: TV (mm^3^) = d^2^xD/2, where d and D are the shortest and the longest diameter, respectively. A tumor growth curve was then obtained.

### Hematoxylin and eosin (H&E) staining

After one month of drug administration, the mice were decapitated and the solid tumor tissue and the lungs were removed. The distribution of tumor cells following drug administration was observed and the tumor metastasis was observed in the lung. Sections of tissue were placed in phosphate-buffered formaldehyde (4%) overnight, then stored in ethanol and embedded in paraffin. Cross sections (4 μm) were stained with H&E.

### Terminal-deoxynucleotidyl transferase-mediated nick end labeling (TUNEL)

Tumor tissue sections fixed in 4% paraformaldehyde accorded to the standard procedure. The slides were placed in a plastic jar containing 200 ml citrate buffer (0.1 M, pH 6.0). Microwave irradiation (750 W) was applied for 1 min. The slides were cooled rapidly by immediately adding 80 ml double distilled water (20–25°C). The slides were transferred into phosphate-buffered saline (PBS; 20–25°C). The slides were then immersed in Tris-HCl (0.1 M; pH 7.5), containing 3% BSA and 20% normal bovine serum for 30 min at 15–25°C. The slides were rinsed twice with PBS at 15–25°C and the excess fluid was drained off. TUNEL reaction mixture (50 ml; Beyotime Institute of Biotechnology, Nantong, China) was added to the sections. For the negative control 50 ml label solution was added. The slides were incubated for 60 min at 37°C in a humidified atmosphere in the dark and then rinsed three times in PBS for 5 min. The sections were analyzed under a fluorescence microscope (model BSF-60; Ba Tuo Instrument Co., Ltd., Shanghai, China) with blue-black particles in the nucleus indicating apoptotic cells.

### Immunohistochemical (IHC) staining

Tumor tissues fixed in 4% paraformaldehyde in PBS were placed on ice for 2 h and saturated in 20% sucrose at 4°C. The samples were embedded in paraffin and cut longitudinally to 4 μm. The sections were washed with PBS and then subjected to staining for VEGF using polyclonal mouse anti-mouse antibodies (BD Biosciences, San Jose, CA, USA) at a dilution of 1:100. The primary antibodies were applied and incubated for 30 min at room temperature. The sections were washed again with PBS and incubated in the biotinylated secondary antibodies (BioSharp, Hefei, China) for 30 min. After three washes with PBS, the sections were incubated in enzyme reagents, containing 50 μl avidin, 50 μl biotinylated horseradish peroxidase and 2.5 ml PBS. Subsequently, the sections were incubated in a few drops of peroxidase substrate mixing liquid and were washed in deionized water. Finally, sections were counterstained in hematoxylin followed by several washes with deionized water. In negative controls, nonimmune serum was used instead of primary antibodies.

## Results

### Tumor growth curve in C_3_H mice

**Figure 1:**
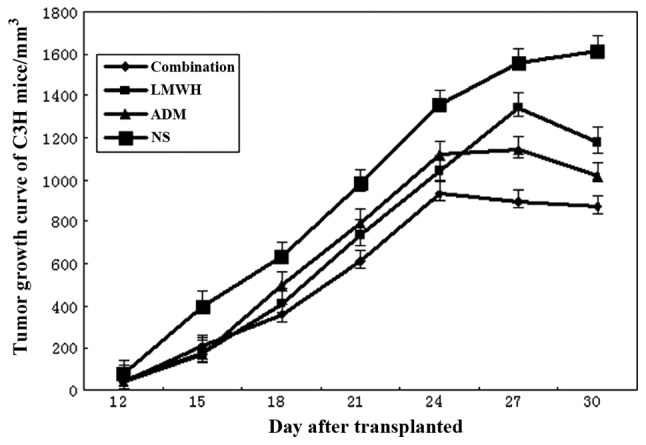
Tumor growth curves in C_3_H mice. LMWH, low molecular weight heparin; ADM, Adriamycin; NS, normal saline.

Twelve days following the inoculation, no significant difference in tumor volume was observed between the four groups. In the normal saline group, as time progressed, the tumor volume increased with a linear upward trend. However, after 27 days, growth slowed. In the LMWH group, after 15 days the tumor volume was similar to that in with the Adriamycin group and combination group, and then increased linearly with time. After day 27, the volume began to decrease; however, it was still higher compared with that in the Adriamycin and combination groups. In the Adriamycin group, the tumor grew rapidly for 24 days, and then the tumor volume gradually declined, but remained higher compared with that in the combination group. In the combination group, after 15 days the volume growth was relatively slow compared with that in the Adriamycin and LMWH groups. After 24 days, the tumor growth began to decline, and the overall volume growth was less compared with that in the other three groups. The tumor growth curves of breast cancer in the four groups of C_3_H mice are shown in Fig. 1.

### H&E staining

**Figure 2:**
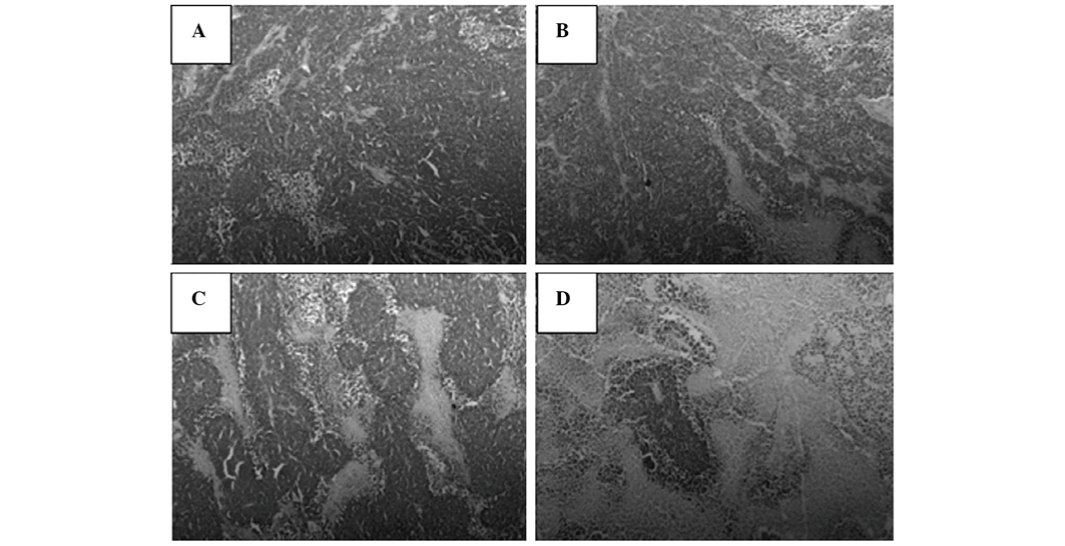
Tumor tissue from the (A) normal saline, (B) low molecular weight heparin, (C) Adriamycin positive control and (D) combination groups (hematoxylin and eosin staining; magnification, ×400).

In the tumor tissue, the tumor cells were found to have a disordered arrangement and a nest-like distribution. The tumor interstitial substance and boundaries were clear, and a slight hyperplasia of fibrous tissue was observed. The number of tumor cells was greater than that of normal cells, and the volume of the tumor nucleus was increased. The nuclei were found to vary in size, shape and coloring, and a number of cases had pathological nuclear fission. These features were most evident in the normal saline group (Fig. 2).

**Figure 3:**
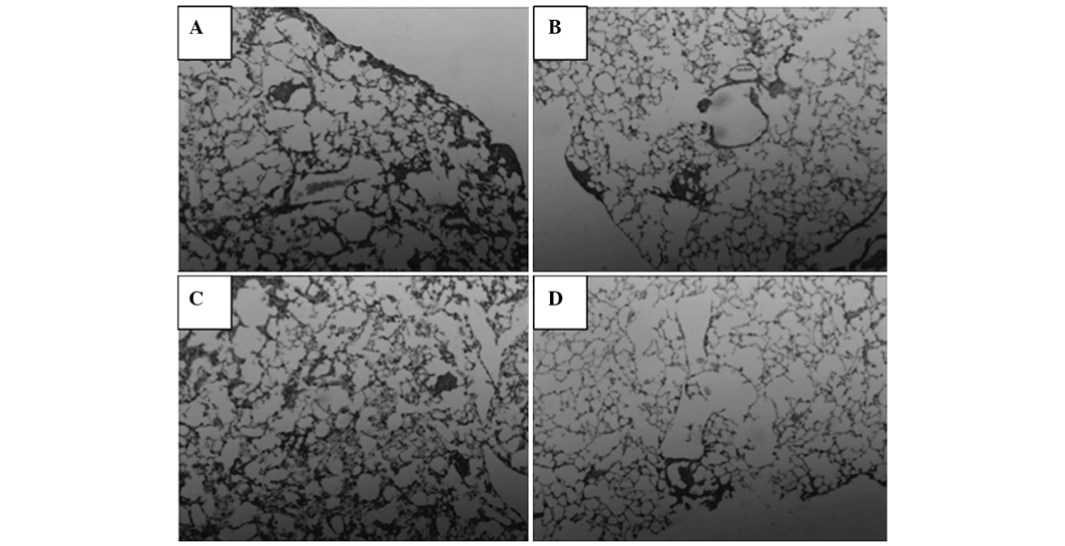
Lung metastasis from the (A) normal saline, (B) low molecular weight heparin, (C) Adriamycin positive control and (D) combination groups (hematoxylin and eosin staining; magnification, ×400).

In the lung, the tumor cells were arranged in nests and strands in the alveolar tissue, accompanied by a small amount of adenoid material. The tumor cells showed marked pleomorphism, and mitotic cells could be observed around the tumor mass, among the fibrous tissue hyperplasia. The normal saline, LMWH and adriamycin groups were observed to have less pleomorphism, mitosis or fibrous tissue hyperplasia compared with the combination group (Fig. 3).

### TUNEL results

**Figure 4:**
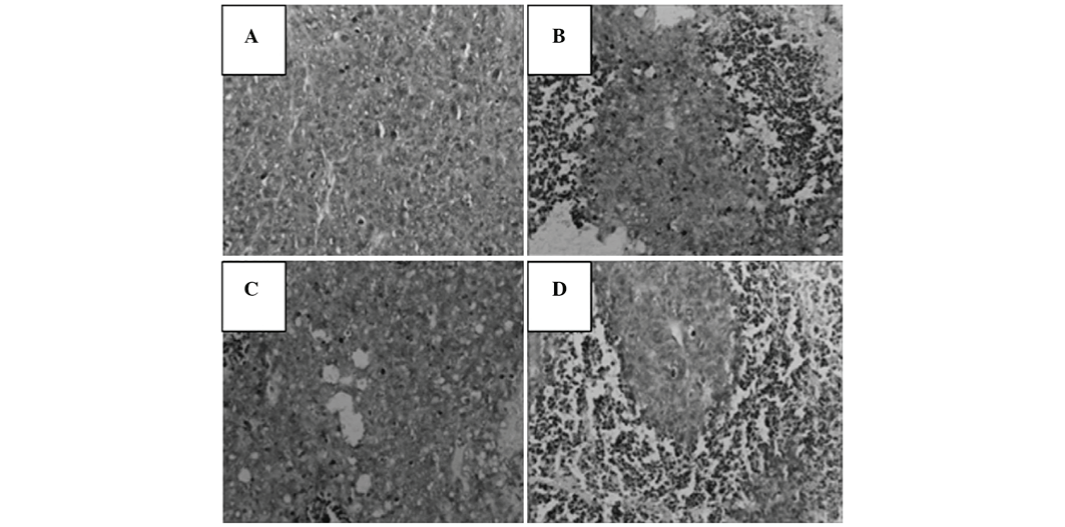
Results of the terminal-deoxynucleotidyl transferase-mediated nick end labeling assay for the (A) normal saline, (B) low molecular weight heparin, (C) Adriamycin positive control and (D) combination groups (magnification, ×400).

TUNEL-positive apoptotic cells showed small condensed nuclei and a circumscribed nuclear membrane, and the nucleus was stained brown. A number of studies have demonstrated that heparin induces the apoptosis of tumor cells (8–10), which is in accordance with the results observed in the present study. The TUNEL results demonstrated that following treatment, the tumor cells became apoptotic, in particular in the combination group (Fig. 4).

### Immunohistochemical results for VEGF

**Figure 5:**
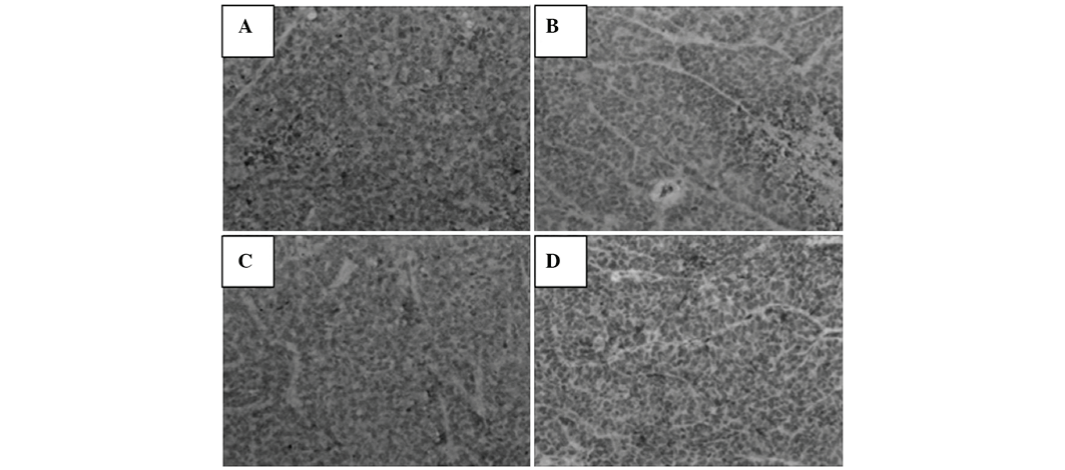
Immunohistochemical results demonstrating the expression of vascular endothelial growth factor in the (A) normal saline, (B) low molecular weight heparin, (C) Adriamycin positive control and (D) combination groups (magnification, ×400).

The immunohistochemical results demonstrated that cytoplasmic expression of VEGF was present in the tumor tissue. Heparin treatment inhibited the expression of VEGF, and the lowest levels of expression were observed in the combination group (Fig. 5).

## Discussion

Breast cancer is the most common malignant tumor in females (11). At present, treatments focus primarily on tumor cells. However, this approach is changing, particularly since numerous studies over the past two decades have demonstrated that cancer is a complex with a large number of components affecting tumor growth, invasion and metastasis. Previous studies have suggested that heparin, as well as having direct effects on blood coagulation, also has a positive effect for the treatment of cancer by inhibiting tumor cell proliferation, angiogenesis, invasion and migration, and enhancing the sensitivity of tumor cells to chemotherapeutic drugs (3–5).

LMWH has certain pharmacokinetic advantages over unfractionated heparin, including a longer half-life, better bioavailability, lower binding to plasma proteins, no requirement for regular laboratory control and the possibility of self treatment at home (12). The results of preclinical and clinical studies have suggested that LMWH inhibits cell growth, cell invasion and angiogenesis in cancer, indicating its anticoagulant and direct antitumor effects (13,14). However, LMWH exhibits a limited single-agent activity, due to hemorrhage and thrombocytopenia in the clinical setting, thus requiring combination with other agents to achieve therapeutic effects (15). Therefore, Adriamycin was selected since it has a broad-spectrum antitumor effect.

The extracellular matrix (ECM) and basement membrane provides an essential physical barrier between cells and tissues, as well as a scaffold for cell growth, migration and differentiation. Studies of ECM molecules in cell attachment, growth and differentiation have indicated that heparan sulfate (HS) proteoglycans are centrally involved in embryogenesis, angiogenesis and epithelial mesenchymal interactions (16,17). HS chains interact with numerous proteins and ensure that a wide variety of bioactive molecules bind to the ECM (18). Heparanase is an endoglucuronidase that cleaves HS, and the expression levels of this enzyme correlate with the metastatic potential of tumor cells (19). LMWH competes with heparanase for the HS acceptor, reducing the degradation of HS and thereby maintaining an intact ECM and inhibiting infiltration and metastasis by the tumor. The results of H&E staining of the lung tissue demonstrated that heparin treatment is able to reduce tumor cell metastasis (Fig. 3) and the TUNEL assay indicated the ability of heparin to induce apoptosis (Fig. 4). This has previously been demonstrated in a number of studies which have shown that heparin reduces tumor metastasis rates by inhibiting heparanase (20,21).

Tumor angiogenesis has an important role in tumor growth and metastasis. The formation of tumor angiogenesis is driven by microvascular endothelial cells (MVECs). The activation of MVECs degrades the basement membrane and allows endothelial cells into the interstitial matrix, where they proliferate and form capillary-like tubular structures. VEGF has a major role in angiogenesis by acting via tyrosine kinase receptors. VEGF antagonists affect tumor growth and vascularization, and the VEGF-specific antibody bevacizumab exerts antivascular effects in patients with cancer (22). LMWH may also inhibit the hyperplasia of endothelial cells and competitively bind to the heparin acceptor, thus affecting angiogenesis factors, particularly VEGF (13,23). The efficacy of an anti-VEGF antibody to inhibit tumor angiogenesis has been shown in lung cancer and human pediatric sarcoma (8,24,25). The results from the present study further confirm that heparin inhibits tumor angiogenesis by inhibiting the expression of VEGF (Fig. 5) to reduce microvascular density and reduce the formation of tumor blood vessels, thus inhibiting tumor growth, invasion and migration.

In conclusion, LMWH was shown to inhibit the growth of breast cancer tumors in C_3_H mice, and the mechanism may be associated with the induction of cancer cell apoptosis and inhibition of neovascularization. The results from the present study indicated that LMWH, combined with a chemotherapeutic agent, exerts an antitumor function, and may therefore provide a novel strategy for tumor treatment. However, due to the complexity of structure and function of heparin, further investigation is required.

## References

[1] Marsden CG, Wright MJ, Carrier L (2012). A novel in vivo model for the study of human breast cancer metastasis using primary breast tumor-initiating cells from patient biopsies. BMC Cancer.

[2] American Cancer Society (2009). Cancer Facts and Figures.

[3] Borsig L (2010). Antimetastatic activities of heparins and modified heparins. Experimental evidence. Thromb Res.

[4] Mousa SA (2010). Heparin and low-molecular weight heparins in thrombosis and beyond. Methods Mol Biol.

[5] Phillips PG, Yalcin M, Cui H (2011). Increased tumor uptake of chemotherapeutics and improved chemoresponse by novel non-anticoagulant low molecular weight heparin. Anticancer Res.

[6] Teoh ML, Fitzgerald MP, Oberley LW, Domann FE (2009). Overexpression of extracellular superoxide dismutase attenuates heparanase expression and inhibits breast carcinoma cell growth and invasion. Cancer Res.

[7] Liu H, Tong X, Ma L, Xu Z (2005). Establishment of a non-spontaneous breast cancer model in C3H mice. Chinese Pharmacological Bulletin.

[8] Dredge K, Hammond E, Handley P, Gonda TJ, Smith MT, Vincent C (2011). PG545, a dual heparanase and angiogenesis inhibitor, induces potent anti-tumour and anti-metastatic efficacy in preclinical models. Br J Cancer.

[9] Li JP (2008). Heparin, heparin sulfate and heparanase in cancer: remedy for metastasis?. Anticancer Agents Med Chem.

[10] Li Y, Liu H, Huang YY, Pu LJ, Zhang XD, Jiang CC, Jiang ZW (2013). Suppression of endoplasmic reticulum stress-induced invasion and migration of breast cancer cells through the downregulation of heparanase. Int J Mol Med.

[11] Reisfeld RA (2013). The tumor microenvironment: a target for combination therapy of breast cancer. Crit Rev Oncog.

[12] Takeuchi A, Yamamoto Y, Munesue S, Harashima A (2013). Low molecular weight heparin suppresses receptor for advanced glycation end products-mediated expression of malignant phenotype in human fibrosarcoma cells. Cancer Sci.

[13] Nagy Z, Turcsik V, Blaskó G (2009). The effect of LMWH (Nadroparin) on tumor progression. Pathol Oncol Res.

[14] Tan H, Yang S, Liu C, Cao J, Mu G, Wang F (2012). Enhanced anti-angiogenesis and anti-tumor activity of endostatin by chemical modification with polyethylene glycol and low molecular weight heparin. Biomed Pharmacother.

[15] Jayson GC, Hicklin DJ, Ellis LM (2012). Antiangiogenic therapy - evolving view based on clinical trial results. Nature Rev Clin Oncol.

[16] Zcharia E, Jia J, Zhang X (2009). Newly generated heparanase knock-out mice unravel co-regulation of heparanase and matrix metalloproteinases. PLoS One.

[17] Kim SH, Turnbull J, Guimond S (2011). Extracellular matrix and cell signaling: the dynamic cooperation of integrin, proteoglycan and growth factor receptor. J Endocrinol.

[18] Lindahl U, Li JP (2009). Interactions between heparan sulfate and proteins - design and functional implications. Int Rev Cell Mol Biol.

[19] Vlodavsky I, Beckhove P, Lerner I, Pisano C, Meirovitz A, Ilan N, Elkin M (2011). Significance of heparanase in cancer and inflammation. Cancer Microenviron.

[20] Basappa, Sugahara K, Thimmaiah KN, Bid HK, Houghton PJ, Rangappa KS (2012). Anti-tumor activity of a novel HS-mimetic-vascular endothelial growth factor binding small molecule. PLoS One.

[21] Battinelli EM, Markens BA, Kulenthirarajan RA, Machlus KR, Flaumenhaft R, Italiano JE (2014). Anticoagulation inhibits tumor cell-mediated release of platelet angiogenic proteins and diminishes platelet angiogenic response. Blood.

[22] Jeong KW, Jeong MC, Jin B, Kim Y (2013). Relationship between structural flexibility and function in the C-terminal region of the heparin-binding domain of VEGF165. Biochemistry.

[23] Jia W, Feng K, Fan P (2012). Post-TACE combination therapy of heparin and octreotide results in decreased tumor metastasis in extrahepatic tumorigenesis. Cell Biochem Biophys.

[24] Shafat I, Bern-Arush MW, Issakov J, Meller I, Naroditsky I, Tortoreto M (2011). Preclinical and clinical significance of heparanase in Ewing’s sarcoma. J Cell Mol Med.

[25] Judy BF, Aliperti LA, Predina JD, Levine D (2012). Vascular endothelial-targeted therapy combined with cytotoxic chemotherapy induces inflammatory intratumoral infiltrates and inhibits tumor relapses after surgery. Neoplasia.

